# The Early Presentation of a Classic Case of Tuberous Sclerosis: A Case Report

**DOI:** 10.7759/cureus.47241

**Published:** 2023-10-17

**Authors:** Shraddha Sawhney, Keta Vagha, Shantanu Gomase, Sham Lohiya, Priyanka Hampe, Ayesha A Ansari, Jayant D Vagha, Anshul Sood

**Affiliations:** 1 Department of Pediatrics, Jawaharlal Nehru Medical College, Datta Meghe Institute of Higher Education and Research, Wardha, IND; 2 Department of Radiology, Jawaharlal Nehru Medical College, Datta Meghe Institute of Higher Education and Research, Wardha, IND

**Keywords:** case report, epilepsy, ash leaf spots, rhabdomyoma, tuberous sclerosis

## Abstract

Tuberous sclerosis (TS) is a potentially severe medical disorder that poses a life-threatening risk and can lead to drastic lifestyle changes. In infants and young children, the typical diagnostic criteria for this condition encompass cutaneous manifestations and seizures, and the development of cellular growths termed hamartomas, astrocytomas, myolipomas, and even carcinomas observed within the cardiac, cerebral, renal, and retinal tissues. The usual age of presentation varies widely, which affects the prognosis. We report a case of a four-month-old male patient who presented with early signs of TS. The patient showed signs of infantile spasms and seizures. On further examination, he had neurological, cutaneous, cardiac, and retinal manifestations, which pointed toward the diagnosis of TS. This case report emphasizes the importance of screening for TS at an early age due to the possibility of patients presenting earlier than the usual age of presentation. To the best of our knowledge, there is scarce data on this kind of early-onset signs of TS; therefore, we feel that it is imperative to start screening infants earlier to improve the prognosis and decrease the complications of this disease. The screening tests and the incidence of screening will vary based on the cost and availability of proper diagnostic and screening tests and the accessibility of efficient treatments.

## Introduction

Tuberous sclerosis complex (TSC) is a neurocutaneous hereditary disease characterized by a high prevalence of epilepsy and neurodevelopmental issues. The incidence of disease occurrence is one in every 15,000 people globally, and there is no gender or racial predominance associated with this condition [1]. The clinical indications of TSC usually appear after six months of birth, making diagnosis challenging in neonates [2]. The significance of the diagnostic criteria pertaining to TSC is contingent upon both their prevalence and specificity. In infants and young children, the typical diagnostic criteria encompass cutaneous manifestations, convulsive episodes, and the development of cellular growths termed hamartomas, primarily observed within the cardiac, cerebral, renal, and retinal tissue. Empirical evidence has substantiated the existence of distinct physical and radiological manifestations indicative of this complex. However, it is imperative to recognize that the diagnostic standards applicable to tuberous sclerosis (TS) and its associated symptoms exhibit age-dependent nuances [3].

In this report, we discuss the case of a four-month-old male who presented with CNS, cardiac, dermatological, and ophthalmic features of TS. The presentation of symptoms starting at the early age of four months is rare, which prompted us to report this unusual case.

## Case presentation

Patient information

A four-month-old male was brought to the emergency unit of our tertiary care hospital in central India by his family due to abnormal head drops and jerky movements. These movements had started during the early morning hours with head drop episodes and jerky movements of the head. According to the mother, the child was having such episodes two to three times per day, each episode lasting for about 30 seconds, which was spontaneously aborted and was followed by micturition and crying. There was no history of frothing from the mouth, depressed activity, altered sensorium, or limb involvement. He had been born of a non-consanguineous marriage, and the birth history had been uneventful. There was no history of seizures in family members.

Clinical results

**Figure 1:**
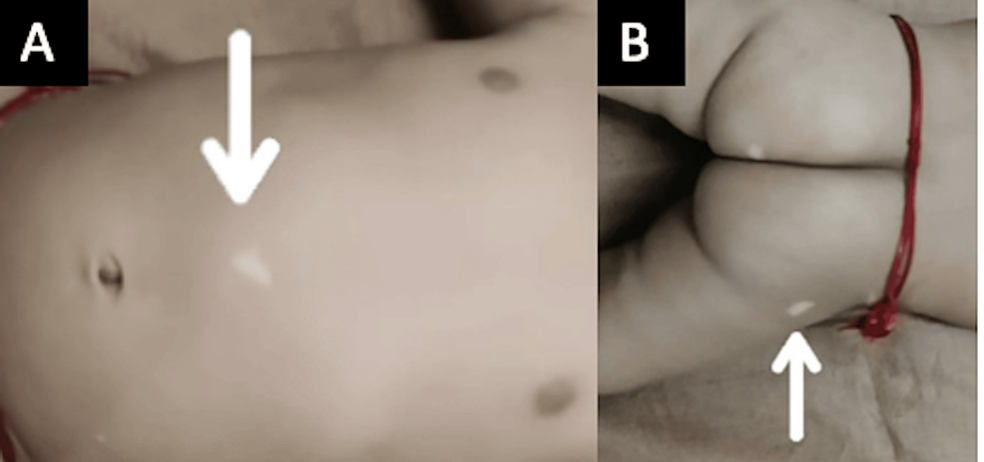
Hypopigmented elongated patches are seen over the trunk (A) and buttocks (B), suggestive of ash leaf macule

On admission, the patient was conscious. His pulse rate was 112 beats per minute, respiratory rate 36 per minute, and SpO_2_ 99 on room air. On general examination, hypomelanotic macules (ash leaf) were seen all over the body, and hence the diagnosis of TS was made (Figure 1).

On systemic examination, there were no systemic abnormalities, and the neurological examination also revealed no apparent abnormality.

Diagnostic approach

**Figure 2:**
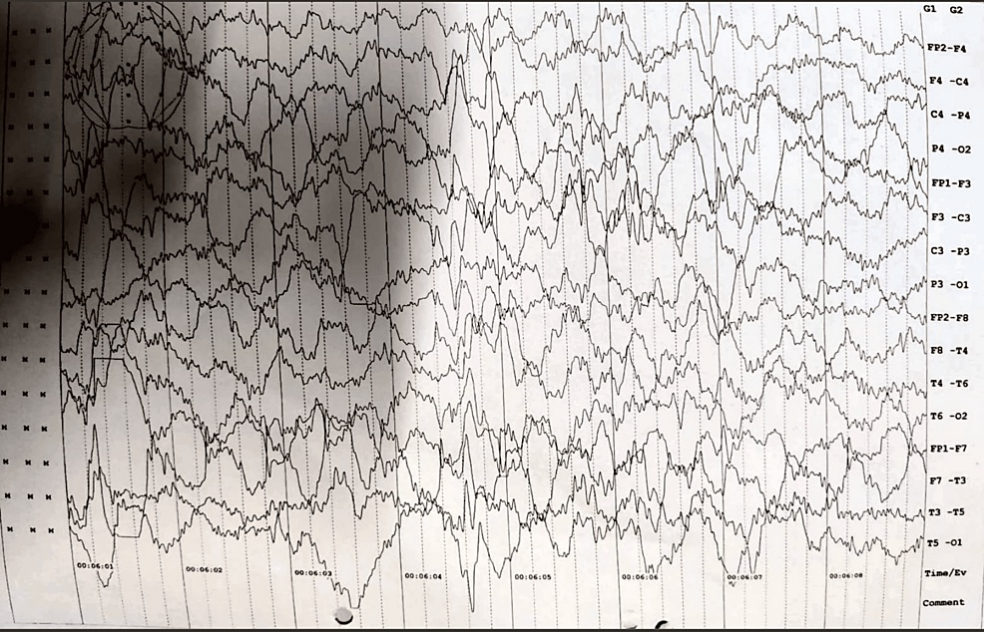
Interictal high amplitude waves and spikes seen in EEG, suggestive of hypsarrhythmia EEG: electroencephalogram

The patient's routine blood investigation results were as follows: hemoglobin: 10.4 gm/dL, total leucocyte count: 12,800/cumm, and platelets: 2.83 lakhs/cumm. His liver function test, coagulation profile, and renal function tests were within normal limits. An EEG was done, which showed chaotic and disorganized brain activity suggestive of hypsarrhythmia (Figure 2).

**Figure 3:**
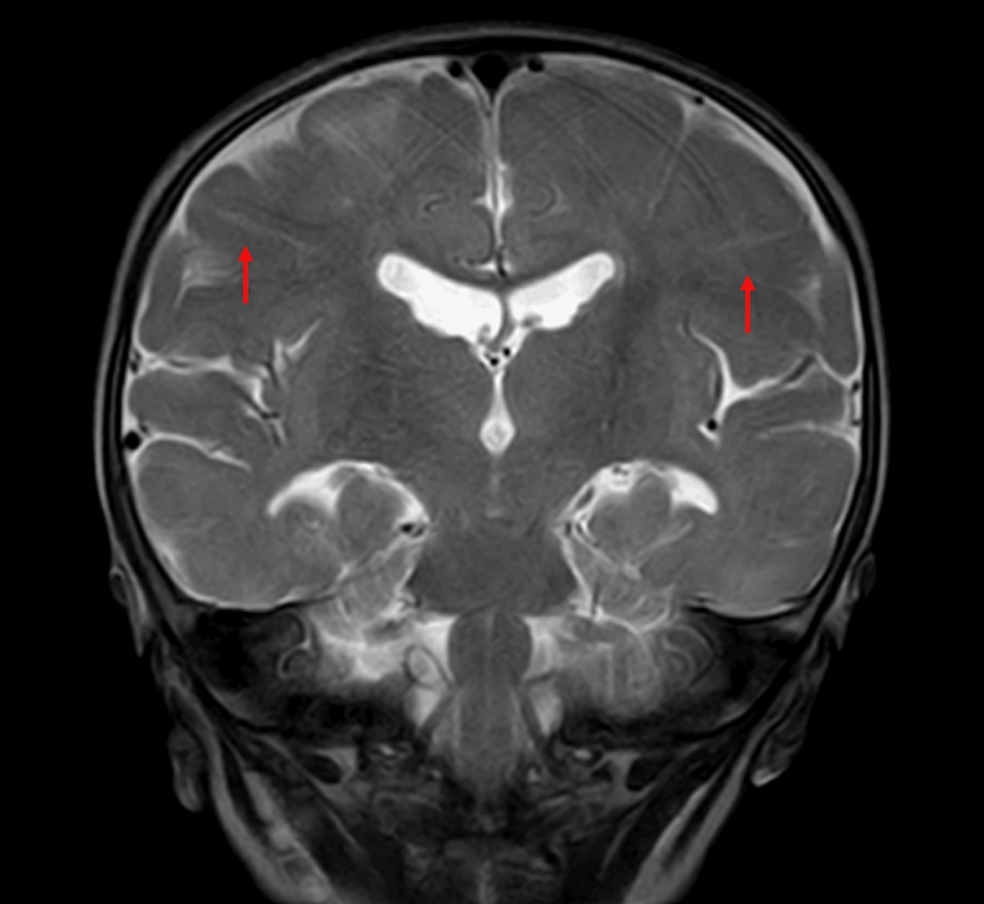
Coronal T2-weighted image showing linear hyperintense bands radiating from periventricular region to cortical surface giving radial band sign

Given the diagnosis of TS, he was screened for the involvement of other organs. A fundus examination was also performed, which was suggestive of astrocytic hamartoma. An MRI brain was done, and multiple radiating linear hyperintense bands (glial bands) were noted in bilateral cerebral hemispheres (radial band sign) (Figure 3).

**Figure 4:**
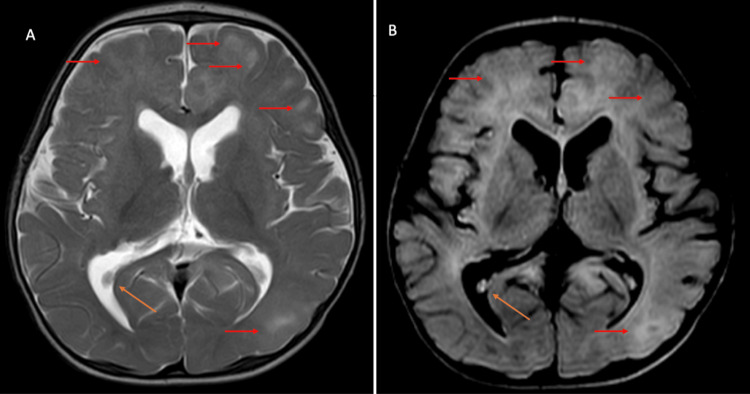
T2-weighted image (A) and fluid attenuation inverse recovery sequence (B) axial sections of the brain showing hyperintensities in the subcortical white matter (red arrows) and subependymal nodule (orange arrow)

Cortical and subcortical tubers were noted in cerebral parenchyma, and multiple subependymal hamartomas were also noted (Figure 4).

**Figure 5:**
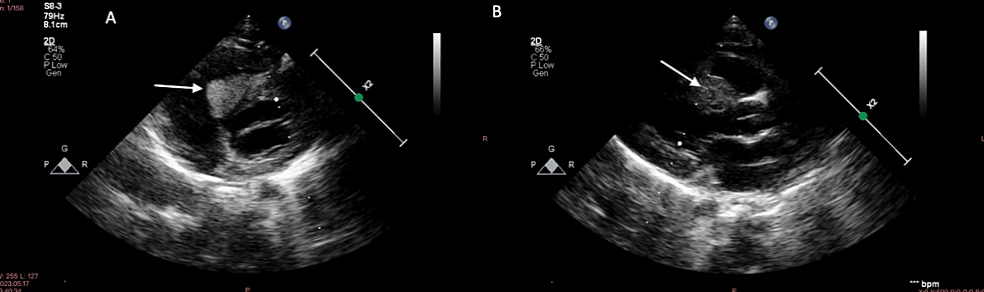
Heterogenous mass present (A and B) in the interventricular septum (IVS) on the right ventricular side

A 2D echo was performed to screen for cardiac manifestations, which showed a heterogenous mass in the interventricular septum on the right ventricular side, suggestive of multiple rhabdomyomas (Figure 5).

Therapeutic intervention

The patient was started on injection ACTH 75 units/m^2^ IM and syrup vigabatrin 50 mg twice a day for hypsarrhythmia and infantile spasms. The dose of vigabatrin was subsequently increased to 100 mg twice per day. He was advised a six-month follow-up for screening of lesions in other organs.

## Discussion

TSC is a disorder with a varied presentation in terms of age-related expression, as well as a variability of clinical manifestations. Due to the complexity of the disease, the clinical diagnosis often poses a challenge. The disease shows an autosomal dominant mode of inheritance and occurs due to a spontaneous genetic mutation seen in either of the two foci of the TSC gene: TSC1 or TSC2 gene [4]. The TSC1 gene is more commonly associated with familial presentations while TSC2 with sporadic. These are tumor suppressor genes that, when mutated, lead to overactivation of the mTOR protein, causing tumor formations in the form of hamartomas [5]. It encompasses various organ systems, notably the CNS (comprising subependymal nodules, cortical hamartomas, and giant cell astrocytoma), the kidneys (involving angiomyolipoma, cysts, and, on rare occasions, renal cell carcinoma), the cardiovascular system (in the form of cardiac rhabdomyoma), the eyes (retinal hamartomas), the skin (featuring hypomelanotic macules, facial angiofibroma, and ungual fibromas), among others [6]. Usually, in the first week of life, children present with a cardiac rhabdomyoma. In the first six months, infantile spasms are seen, and in late infancy, the child presents with new-onset seizures, infantile spasms, and hypopigmented patches. The study by Webb et al. observed that infantile spasms and epilepsy were present in over 78% of patients with TSC in the first year of life [7].

Another study by Kingswood et al. stated that epilepsy is the most commonly reported presentation of TS, with focal seizures being the most common type of seizure, which preceded infantile spasm [8]. These findings are consistent with those in our case. Regarding the median age of occurrence of the symptoms, most patients present at one year, with cardiac rhabdomyoma not presenting until three years of age [9]. However, our patient presented with infantile spasms, cardiac manifestations, cutaneous lesions, and retinal hamartomas at four months. Therefore, early screening and diagnosis of the disease are imperative. In some cases, prenatal screening enables the amelioration of CNS complications such as epilepsy, cognitive and intellectual impairment, and behavioral disorders. Since cardiac rhabdomyoma is the first sign of TS, the fetus should also undergo a thorough neurosonography examination.

As per the International Tuberous Sclerosis Complex Consensus Conference 2012, the clinical diagnostic criteria for identifying TSC encompass certain major and minor features. The major features include the presence of three hypomelanotic macules, each with a diameter of at least 5 mm, and the manifestation of three or more angiofibroma or a fibrous cephalic plaque. Additionally, two or more ungual fibromas, a Shagreen patch, multiple retinal hamartomas, cortical dysplasias, subependymal nodules, subependymal giant cell astrocytoma, cardiac rhabdomyoma, lymphangioleiomyomatosis (LAM), and two or more angiomyolipomas are considered major diagnostic criteria. Conversely, minor criteria include "confetti" skin lesions, the presence of more than three dental enamel pits, two or more intraoral fibromas, a retinal achromic patch, multiple renal cysts, and nonrenal hamartomas. The diagnosis is confirmed if two major features or one major and two or minor features are observed [10]. Our patient presented with two significant features, i.e., hypomelanotic macules, which were more than three in number and greater than 5 mm in size, and cardiac rhabdomyoma. Hence, the diagnosis of TS was made.

Due to the limitations of prenatal genetic testing, the distinction between the causative gene being TSC 1 or 2 could not be identified.

## Conclusions

This clinical report discussed a case of early presentation of TS at four months. The disease may present at an early age in genetically predisposed patients and those who have undergone prenatal screening. Due to the subtlety and complexity of the presenting symptoms, a definitive diagnosis of the disease could be delayed till later in life, resulting in complications and a poor prognosis. However, the presence of infantile spasms, cardiac rhabdomyoma, and ash leaf lesions supported an early diagnosis in our case. Therefore, early screening and investigations are necessary among genetically predisposed patients, and a thorough and detailed history is paramount for appropriate treatment.
